# Editorial: Beyond Histocompatibility – Understanding the Non-MHC Determinants Shaping Transplantation Outcome and Tolerance Induction

**DOI:** 10.3389/fimmu.2021.759706

**Published:** 2021-09-07

**Authors:** Craig A. Byersdorfer, Hēth R. Turnquist

**Affiliations:** ^1^Department of Pediatrics, University of Pittsburgh School of Medicine, University of Pittsburgh, Pittsburgh, PA, United States; ^2^Department of Immunology, University of Pittsburgh School of Medicine, University of Pittsburgh, Pittsburgh, PA, United States; ^3^Thomas E. Starzl Transplantation Institute, University of Pittsburgh School of Medicine, University of Pittsburgh, Pittsburgh, PA, United States; ^4^Department of Surgery, University of Pittsburgh School of Medicine, University of Pittsburgh, Pittsburgh, PA, United States; ^5^McGowan Institute for Regenerative Medicine, University of Pittsburgh School of Medicine, University of Pittsburgh, Pittsburgh, PA, United States

**Keywords:** transplantation, innate and adaptive immune response, damage associated molecular pattern (DAMP), immunometabolism, solid organ transplantation, allogeneic stem cell transplantation, macrophages, GvHD

In seventy years, solid organ transplantation (SOTx) has progressed from a high-risk experimental procedure, with few recipients surviving into the 2nd year post-transplant, to becoming the standard of care for many end-stage organ diseases (1–3). During a similar period, allogeneic stem cell transplantation (AlloHCT) became a central treatment for malignancy and to correct life-threatening lymphohematopoietic disorders (4). This success is due to the development of potent immunosuppressants that blunt T cell responses to major and minor histocompatibility complex (MHC) antigens, the dominant T cell targets after transplantation. Yet, numerous hurdles remain. Toxicities and side effects associated with general immunosuppression are well appreciated. Furthermore, while immunosuppression is a necessary evil after life sustaining SOTx, it is a roadblock for progress in the application of composite tissue allografts (CTA). Here, the side effects from the high doses of immunosuppression needed may outweigh the benefits to life quality provided. In both CTA and SOTx, even toxic levels of immunosuppressants are ineffective against the development of vasculopathy and fibrosis in solid organs over time (5). Finally, the combination of donor AlloHCT and SOTx has provided evidence that tolerance to donor antigens can be induced, yet the risk of graft-versus-host disease (GVHD) and unidentified barriers to routine tolerance induction with these protocols remain (6).

Articles in this Research Topic encompass Original Research and Reviews from transplant researchers seeking to understand the mechanisms controlling transplant outcomes beyond T cell recognition of donor MHC. It is widely accepted that allograft rejection results from coordinated interactions between the innate and the adaptive immune systems, where activated myeloid antigen presenting cells (APCs), such as dendritic cells (DC) or macrophages, stimulate alloreactive T cell responses. A number of the articles received focused on how innate immune cell can also act as direct effectors of transplant outcomes. In one review, we focused on the consolidating knowledge that damage associated molecular patterns (DAMPs) coordinate the function of innate immune cells to shape alloimmunity and rejection, but also direct graft tissue repair (Dwyer and Turnquist). Here, we also made the case that poor outcomes after SOTx may result not only from abundant DAMP-driven inflammation supporting rejection, but also from inadequate or dysregulated DAMP-mediated tissue repair (Dwyer and Turnquist). Similarly, Ordikhani et al. make a convincing argument that macrophages have a dual role in allograft transplantation and can both trigger inflammatory responses or induce tolerogenic environments. They highlight the role of monocytes and macrophages in SOTx, summarize macrophage heterogeneity, and describe the role of macrophages in rejection versus tolerance. In particular, they highlight monocyte “trained immunity” that is associated with augmented immune responses and retained by epigenetic and metabolic changes. They further suggest that therapeutic targeting of trained immunity represents a novel paradigm to prevent allograft rejection. A review by Zhao et al. furthers similar considerations, first by making a strong case for innate immune cell involvement in allograft rejection, followed by a detailed review of how donor polymorphisms in SIRPα promote allorecognition by CD47 signaling on monocytes. This cascade causes accumulation of monocyte-derived DCs and initiation/maintenance of T cell responses. The authors then describe an innate memory response to MHC class I that is driven by paired immunoglobulin-like receptor A (PIR-A) molecules sensing allogeneic MHCI and contributing to SOTx chronic rejection. The authors close their review by contextualizing the impact of innate allorecognition and innate memory in relevant clinical scenarios.

DCs are increasingly part of therapeutic strategies to achieve allograft tolerance. Schroth et al. provide a timely review of emerging roles different DC subsets and their molecular protagonists play during allograft rejection and tolerance after cardiac transplantation. They highlight differential roles for DC subsets, describe the part innate DCs play in cardiac transplantation, and describe the prominence of DCs in tolerance induction therapies using apoptotic donor cells and costimulatory blockade, and the relation of DC immunometabolism to effector phenotype. Rouselle et al. explore this topic in a series of studies testing if DCs propagated *ex vivo* in the presence of FTY720 (FTY), a Sphingosine 1-phosphate receptor (S1PR) agonist, could protect against kidney ischemia reperfusion injury (IRI). Adoptive transfer of FTY-DCs significantly protected kidneys from IRI, a result dependent upon a recipient spleen, DC expression of S1P1, and functional viability of DC-associated mitochondria. Their report further implicates a mechanism involving transfer of mitochondria to splenic macrophages as an underlying mechanism and support this supposition by demonstrating the transfer of mitochondria from bone marrow-derived DCs to cultured macrophages. Molina et al. investigate DC biology in the context of GVHD by demonstrating that pre-transplant conditioning with bendamustine plus total body irradiation increased CD8α cDC1 cell number and percentage, a subset known to ameliorate GVHD (7, 8), and promoted commitment of DC progenitors to the cDC1 lineage pre-transplant, where expression of CD24 allowed enhanced DAMP sensing.

Metabolic reprogramming is critical to T cell activation, differentiation, and function (9). Cheng et al. build on their past work in this space and demonstrate that costimulatory blockade in combination with targeting T cell metabolism can promote skin allograft survival and long-term cardiac allograft acceptance in the absence of maintenance immunosuppression. Interestingly, metabolic inhibition appeared to play more of a role during acute rejection, while addition of CTLA4-Ig demonstrated a synergistic effect on acute and memory T cell responses. Our review (Brown and Byersdorfer) summarizes the current understanding of the metabolic pathways available to alloreactive T cells and highlights key metabolic proteins and pathways linking T cell metabolism to effector function. A current picture of alloreactive T cell metabolism during AlloHSCT is provided, with roles for glycolysis, fat oxidation, and glutamine metabolism as well as a potential explanation for how presumably contradictory metabolic findings might be reconciled. Finally, the caveats and challenges of assigning causality using the current metabolic toolbox, as well as future directions in the field, are summarized.

Other papers in the Research Topic provide novel insights into mechanisms shaping T cell functions after transplantation. Activation of GVHD-causing alloreactive T cells relies on TCR engagement (Signal 1) and coordinated co-stimulation (Signal 2), in concert with signals from a network of secreted cytokines (Signal 3). The review by Kim and Reddy describes current approaches targeting Signal 3 in clinical GVHD and reviews extracellular cytokine blockade, therapies that target intracellular cytokine synthesis, and pathways which impact cytokine transport, the latter of which represents a novel starting point for rational design of GVHD therapies (Kim and Reddy). In their work, Mammadli et al. demonstrate that as well as the intracellular signaling protein Interleukin-2-inducible T cell Kinase (ITK) is necessary in T cells for the development of GVHD but dispensable for graft-versus-leukemia (GVL) effects. Mechanistically, investigators noted a cell intrinsic decrease in proinflammatory cytokine expression and cell extrinsic decrease in CD8 T cell proliferation, as well as impaired chemokine expression in ITK knock-out cells, resulting in decreased migration to target organs. In their review, Gill and Burrack describe two concepts that expand the commonly held view of how memory cells contribute to transplantation immunity and tolerance disruption. First, they stress that autoimmune T cells may interact with graft-derived autoantigens in addition to cross-reactive, heterologous alloimmune MHC molecules. Additionally, they posit that a common APC may license naïve alloreactive T cells if a vaccine- or pathogen-directed memory cells recognizes the same APC *in vivo*. Indeed, this speculation is reminiscent of CD4 helper T cells licensing CD8 cytotoxicity and suggest that assessing only anti-donor MHC reactivity pre-transplant may insufficiently predict success in tolerance-promoting therapies. Understanding transplantation tolerance also requires knowledge of the crosstalk between pathogenic T cells and their tissue resident counterparts. In their review, Lei et al. focus on interactions between alloreactive T cell and cells of the liver microenvironment, paying particular attention to adhesion molecule, chemokine expression, cytokine secretion by immune cells, and a role for regulatory T cells in promotion of transplant-specific tolerance. The authors close with a review of recent and ongoing clinical trials that seek to influence post-transplant tolerance using cell mediated approaches.

The studies presented in this Research Topic show how our knowledge of transplant biology continues to not only increase, but also broaden into new roles for long studied immune cells and immunological mechanisms. There is clearly a rapidly advancing understanding of innate immunity, improved manipulation of immune cell metabolism, and functional elucidation of the pathways controlling pathogenic alloreactive immune responses cells in SOTx and AlloHCT. There has been a sustained history of observation, collaboration, and knowledge assimilation between research discoveries and clinician scientists in the fields of SOTx and AlloHCT. It is encouraging to see investigators in both areas in this Research Topic, and we hope that efforts like this help the diverse groups of transplant scientists learn from each other as we aim to provide transplant recipients with the best hope for long-term resolution with the least amount of toxicity.

## Author Contributions

Both authors generated and edited the editorial. All authors contributed to the article and approved the submitted version.

## Funding

The authors are supported by NIH grants: R01AR073527 (HT), R01HL22489 (HT), R56AI139327 (HT), and R01HL144556 (CB), DoD grant CA180681 (CB) and a Hope on Wheels Scholar grant from the Hyundai Motor Company (CB).

## Conflict of Interest

The authors declare that the research was conducted in the absence of any commercial or financial relationships that could be construed as a potential conflict of interest

## Publisher’s Note

All claims expressed in this article are solely those of the authors and do not necessarily represent those of their affiliated organizations, or those of the publisher, the editors and the reviewers. Any product that may be evaluated in this article, or claim that may be made by its manufacturer, is not guaranteed or endorsed by the publisher.
